# Predicting the pathogenicity of missense variants using features derived from AlphaFold2

**DOI:** 10.1093/bioinformatics/btad280

**Published:** 2023-04-21

**Authors:** Axel Schmidt, Sebastian Röner, Karola Mai, Hannah Klinkhammer, Martin Kircher, Kerstin U Ludwig

**Affiliations:** Institute of Human Genetics, Bonn School of Medicine, University Hospital of Bonn, University of Bonn, Bonn, Germany; Berlin Institute of Health at Charité—Universitätsmedizin Berlin, Berlin, Germany; Institute of Human Genetics, Bonn School of Medicine, University Hospital of Bonn, University of Bonn, Bonn, Germany; Institute for Genomic Statistics and Bioinformatics, University Hospital of Bonn, University of Bonn, Bonn, Germany; Institute of Medical Biometry, Informatics and Epidemiology, University Hospital of Bonn, University of Bonn, Bonn, Germany; Berlin Institute of Health at Charité—Universitätsmedizin Berlin, Berlin, Germany; Institute of Human Genetics, University Medical Center Schleswig-Holstein, University of Lübeck, Lübeck, Germany; Institute of Human Genetics, Bonn School of Medicine, University Hospital of Bonn, University of Bonn, Bonn, Germany

## Abstract

**Motivation:**

Missense variants are a frequent class of variation within the coding genome, and some of them cause Mendelian diseases. Despite advances in computational prediction, classifying missense variants into pathogenic or benign remains a major challenge in the context of personalized medicine. Recently, the structure of the human proteome was derived with unprecedented accuracy using the artificial intelligence system AlphaFold2. This raises the question of whether AlphaFold2 wild-type structures can improve the accuracy of computational pathogenicity prediction for missense variants.

**Results:**

To address this, we first engineered a set of features for each amino acid from these structures. We then trained a random forest to distinguish between relatively common (proxy-benign) and singleton (proxy-pathogenic) missense variants from gnomAD v3.1. This yielded a novel AlphaFold2-based pathogenicity prediction score, termed AlphScore. Important feature classes used by AlphScore are solvent accessibility, amino acid network related features, features describing the physicochemical environment, and AlphaFold2’s quality parameter (predicted local distance difference test). AlphScore alone showed lower performance than existing *in silico* scores used for missense prediction, such as CADD or REVEL. However, when AlphScore was added to those scores, the performance increased, as measured by the approximation of deep mutational scan data, as well as the prediction of expert-curated missense variants from the ClinVar database. Overall, our data indicate that the integration of AlphaFold2-predicted structures can improve pathogenicity prediction of missense variants.

**Availability and implementation:**

AlphScore, combinations of AlphScore with existing scores, as well as variants used for training and testing are publicly available.

## 1 Introduction

The comprehensive assessment of genetic variation using exome or genome sequencing to identify variants causing monogenic diseases is becoming increasingly routine in clinical genetics. Variants considered are nowadays typically assessed using the criteria of the American College of Medical Genetics and Genomics (ACMG criteria) (Richards et al. 2015) or refinements based on these. In the ACMG criteria, variants are classified into one of five pathogenicity classes [benign, likely benign, variant of uncertain significance (VUS), likely pathogenic, and pathogenic] based on weighted sums of arguments for and against a possible pathogenicity. Arguments considered include predicted functional effect, segregation, and frequency data, as well as computational prediction scores.

While loss-of-function variants are comparably easy to interpret, the assessment of missense variants is challenging and the application of the ACMG criteria often results in the classification VUS, which is of limited benefit to the patient and their clinical care. At the time of writing, 92% of missense variants (190 258 of 205 726) in ClinVar were classified as VUS or variants with conflicting interpretations of pathogenicity. In contrast, this proportion was only 13% for frameshift variants (3136 of 24 122) (Landrum et al. 2018). The challenge of classifying missense variants is additionally aggravated by the fact that rare missense variants are relatively frequent in individual genomes (Karczewski et al. 2020). Therefore, additional approaches are needed to better classify missense variants in the future such as improved computational prediction scores.

One widely used computational prediction score is the Combined Annotation Dependent Depletion (CADD) score, which was first described in 2014 (Kircher et al. 2014) and has seen major updates since then (Rentzsch et al. 2019). CADD integrates a wide range of features, such as the missense prediction scores SIFT (Ng and Henikoff 2003) and PolyPhen-2 (Adzhubei et al. 2010). Recently, two additional computational prediction scores that yield missense variant-specific scores have increased in popularity: (i) REVEL, an ensemble method that combines 13 established prediction tools (including PolyPhen-2 and SIFT; Ioannidis et al. 2016) and (ii) DEOGEN2 (Raimondi et al. 2017), which incorporates information concerning the gene, its protein domains, and interactions. Benchmarks have shown that REVEL and DEOGEN2 predictions correlate well with deep mutational scan (DMS) data (Livesey and Marsh, 2020).

So far, none of the commonly used prediction scores systematically includes information of the 3D protein structure. This may be attributable, in part, to the fact that for ∼80% of residues no experimental structures are yet available (Somody et al. 2017; Diwan et al. 2021). First attempts to incorporate protein information have been performed by Polyphen-2, which uses information on accessible surface area of the residue, change in hydrophobic propensity, and the crystallographic B-factor, for those proteins where experimental protein structures were available. Notably, 1 of the 11 features used in DEOGEN2 is “protein–protein interaction” as derived from experimental protein structures.

Recently, the artificial intelligence system AlphaFold2 generated a highly accurate prediction of nearly all 3D protein structures of the human proteome (Jumper et al. 2021; Tunyasuvunakool et al. 2021). It has been suggested that the AlphaFold2 system cannot directly predict the effect of missense variants (Buel and Walters 2022). However, AlphaFold2 wild-type structures have already been used to generate a constraint score of individual amino acids by integrating 3D structural information (7020 of the 16 533 structures used; Li et al. 2022), and to predict the effect of missense variants on protein stability (Akdel et al. 2022; Chuah et al. 2022; Iqbal et al. 2022). Still, the potential of AlphaFold2-derived protein structures for improving the *in silico* prediction of the pathogenicity of missense variants in the human genetics context is only partially explored.

The aim of the present study was to determine whether the structure predictions of AlphaFold2 can improve systematic prediction of the pathogenicity of missense variants. For this purpose, we extracted a set of features from the predicted structures to train tree-based machine-learning classifiers. Using variants from DMS and ClinVar, we show that AlphaFold2 structures contain additional information that is beneficial for pathogenicity assessment, and that this information can be integrated with existing prediction scores in order to increase their predictive value.

## 2 Methods

### 2.1 Software

The present data were generated using a custom Snakemake pipeline, which is available via github (https://github.com/Ax-Sch/AlphScore). The main frameworks used were Snakemake version 6.12.3, Python version 3.10, and R version 4.1.1.

### 2.2 Datasets

For training and testing, two missense variant datasets were defined. The first contained missense variants derived from the population database gnomAD [version 3.1 (Karczewski et al. 2020) as included in dbNSFP]. A flowchart describing the creation of this dataset is shown in Supplementary Fig. S2A. Variants in this dataset with an allele frequency >0.1% were labeled as proxy-benign. In contrast, proxy-pathogenic variants were defined as singleton variants in gnomAD genomes that originated from the Non-Finnish European (NFE) subcohort and which were absent in both the 1000 Genomes Project and the National Heart, Lung, and Blood Institute (NHLBI) Exome Sequencing Project (ESP6500). Due to the potential sample overlaps between gnomAD genomes (version 3.1) and gnomAD exomes (version 2.1.2), in gnomAD exomes an allele count of 1 was tolerated if variants originated from the NFE subcohort of gnomAD exomes. To reduce biases in the training set, the proxy-pathogenic variants were subsampled to yield a constant ratio of proxy-pathogenic to proxy-benign variants for each reference amino acid (see Supplementary Fig. S3, ratio approximately 0.78).

The second dataset was created by filtering ClinVar (version 20210131 as included in dbNSFP, Landrum et al. 2018) for missense variants labeled as benign/likely benign or pathogenic/likely pathogenic.

To split training and validation sets, 80% of proteins were selected at random. The gnomAD variants within these proteins served as the training set (gnomAD_train), whereas variants that were present in ClinVar but not in gnomAD_train served as the validation set (ClinVar_val). ClinVar variants within the remaining 20% of proteins, and ClinVar variants that were new to ClinVar version 20220109 (downloaded from NCBI, Landrum et al. 2018), were used as the final test set (ClinVar_test). The independence of the datasets was confirmed by mutual exclusion of variants via chromosome, position, reference allele, and alternative allele (see Supplementary Fig. S1).

### 2.3 Feature extraction

Protein structures predicted by AlphaFold2 for the human reference proteome were downloaded from the EMBL-EBI website (https://alphafold.ebi.ac.uk/download, 29 November 2021, version 2). Several tools were applied for feature extraction. First, secondary structures and solvent-accessible surface areas were extracted using the software DSSP [version 3.0.0 (Kabsch and Sander 1983)]. Second, the FEATURE framework [version 3.1.0 (Halperin et al. 2008)] was applied to calculate physicochemical features within spheres with diameters of 0, 3, and 6 angstroms. The values of the C-alpha atom of the FEATURE framework were selected for each residue. Inter-residue interactions and contacts were also extracted using a modified version of the Protinter software (https://github.com/Ax-Sch/protinter). Half-sphere exposures and the predicted local distance difference test (pLDDT) were extracted using the Biopython or the Biopandas python package, respectively. Finally, a weighted amino acid network was constructed for each protein using the python package biographs and an atom–atom distance cutoff of 4 Å (https://github.com/rodogi/biographs). This network was used to calculate weighted means of the properties of neighbor amino acids, as well as network-based metrics, using the python package networkx (see Supplementary Table S1). In cases where several structural models were available for one protein, the average value for each feature across the structural models was used. The features obtained for each amino acid were then added to the dbNSFP4.2a database (Liu et al. 2011, 2020) using the UniProt ID and the Variant Effect Predictor annotations as keys.

### 2.4 Data preprocessing

In addition to the features described above, 20 binary variables encoding the reference amino acid and 20 binary variables representing the alternative amino acid were created (see Supplementary Table S1). To optimize representation of the effects of amino acid substitutions, the average values of selected features for each of the 20 possible amino acids were determined. For this purpose, the gnomAD_train dataset was used and filtered for residues with high confidence prediction (pLDDT > 90). The average value obtained was attributed to the alternative amino acid for each missense variant. The difference between this average value assigned to the alternative amino acid and the value extracted for the reference amino acid was then calculated. This difference was appended to the dataset.

### 2.5 Machine learning and grid search

Due to the robustness and interpretability of tree-based predictors, gradient boosting (R package xgboost), random forests (R package ranger), and extremely randomized trees (R package ranger) were selected as candidate algorithms. Several algorithm/parameter combinations were evaluated during the grid search, as shown in Supplementary Table S3. The performance of each model was evaluated in ClinVar_val by calculating the area under the receiver operating characteristics (AUROC). The best model (AlphScore) used random forests, as implemented in the package ranger with 2000 trees (num.trees), a maximal depth (max.depth) of 5, and a minimal node size (min.node.size) of 10. Otherwise, default parameters were used. Prior to model fitting, identical features were pruned (correlation higher than 0.999999). The features listed in Supplementary Table S1 were used for prediction.

In addition, we investigated whether changes to the filter criteria for the proxy-pathogenic variants of the training set would influence the results. Therefore, the filters based on the 1000 Genomes Project and the NHLBI Exome Sequencing Project (ESP6500) were omitted (see above and see Supplementary Fig. S2A) and a new model was generated with the modified training set, using the same procedures as described for AlphScore. The Spearman correlation between the model scores obtained in this way and AlphScore was then calculated on independent ClinVar variants (ClinVar version 20210131; cor.test command in R, see Supplementary Fig. S2B).

### 2.6 Analysis of model characteristics

To calculate permutation-based feature importance for AlphScore, the option importance=“permutation” was set in the package ranger during model fitting. The permutation procedure is described in detail on page 3 of Wright et al. (2016). In short, for each feature the values are permuted between observations; the drop in predictive performance is then used as an indicator of feature performance. To assess the relevance of certain groups of features, two additional models were fitted. These models used the parameters of the top performing model, with the exception that either all AlphaFold-derived features (NullModel) or all features containing AlphaFold’s pLDDT parameter were removed from the provided predictor variables.

### 2.7 Combination with other missense prediction scores

AlphScore was combined with established missense prediction scores using logistic regression as implemented in the R-function glm with the option family=binomial(link=“logit”). The validation dataset ClinVar_val was used as the source of training data.

### 2.8 DMS data and evaluation

DMS data for the following proteins were obtained from the supplementary table provided by Livesey and Marsh (2020): UBE2I, SUMO1, TPK1 (Weile et al. 2017); BRCA1 (Starita et al. 2015; Findlay et al. 2018); P53 (Giacomelli et al. 2018); HRAS (Bandaru et al. 2017); PTEN (Matreyek et al. 2018; Mighell et al. 2018); and ADRB2 (Jones et al. 2020). DMS data for MSH2 and VKOR1 were added (Chiasson et al. 2020; Jia et al. 2021). In cases where a study had conducted different experiments under different conditions, the condition with the highest overall absolute correlation to the computational predictors was used. When DMS data for <20 variants were available for any computational score, the respective DMS dataset was excluded. This filter led to the exclusion of the protein CALM1 from Weile et al. (2017). To ensure comparability, variants of DMS datasets for which values from one of the prediction scores were missing were excluded. Spearman correlations between the DMS scores and the computational scores were calculated using the cor.test command in R. In the case of negative correlations, the absolute value was reported. To assess score performance, we calculated mean absolute Spearman correlation with the 13 DMS datasets. Additionally, we calculated rank score statistics as previously described in Livesey and Marsh, 2020.

### 2.9 Bootstrapping

To estimate the uncertainty in our results, we used bootstrapping. We generated 1000 bootstrapped samples from the variants for which DMS data were available and from the ClinVar_test dataset, respectively, using the R package boot with default parameters. In each of these samples, we then calculated rank score statistics (DMS data) or AUROCs (ClinVar_val).

### 2.10 Fitting the final model (AlphScore_final)

The parameters of the best performing prediction model were used to fit a model on the full set of gnomAD variants (not restricted to certain genes). This model was then combined with CADD, REVEL, and DEOGEN2 using logistic regression and all (likely) benign/(likely) pathogenic ClinVar variants that were not contained within the training set (as described above). The final model was evaluated with ClinVar variants that were new in ClinVar version 20220109 to ensure that this model had similar performance to AlphScore (see Supplementary Figs S9 and S10).

### 2.11 Comparison of variants in membrane- and non-membrane-associated proteins

Membrane-associated proteins were defined using the PANTHER database (version 17.0; Thomas et al. 2003). In PANTHER protein classes, the name and description was searched for “membrane,” “junction,” or “transporter.” The protein classes thus obtained were manually reviewed and, in the next step, proteins annotated with these protein classes were collected (see Supplementary Table S5). The ClinVar_test dataset was then split into two parts—variants in proteins that are membrane-associated according to this definition and all other variants. We then calculated AUROCs as described above.

### 2.12 Generation of figures

**Figure 1.**
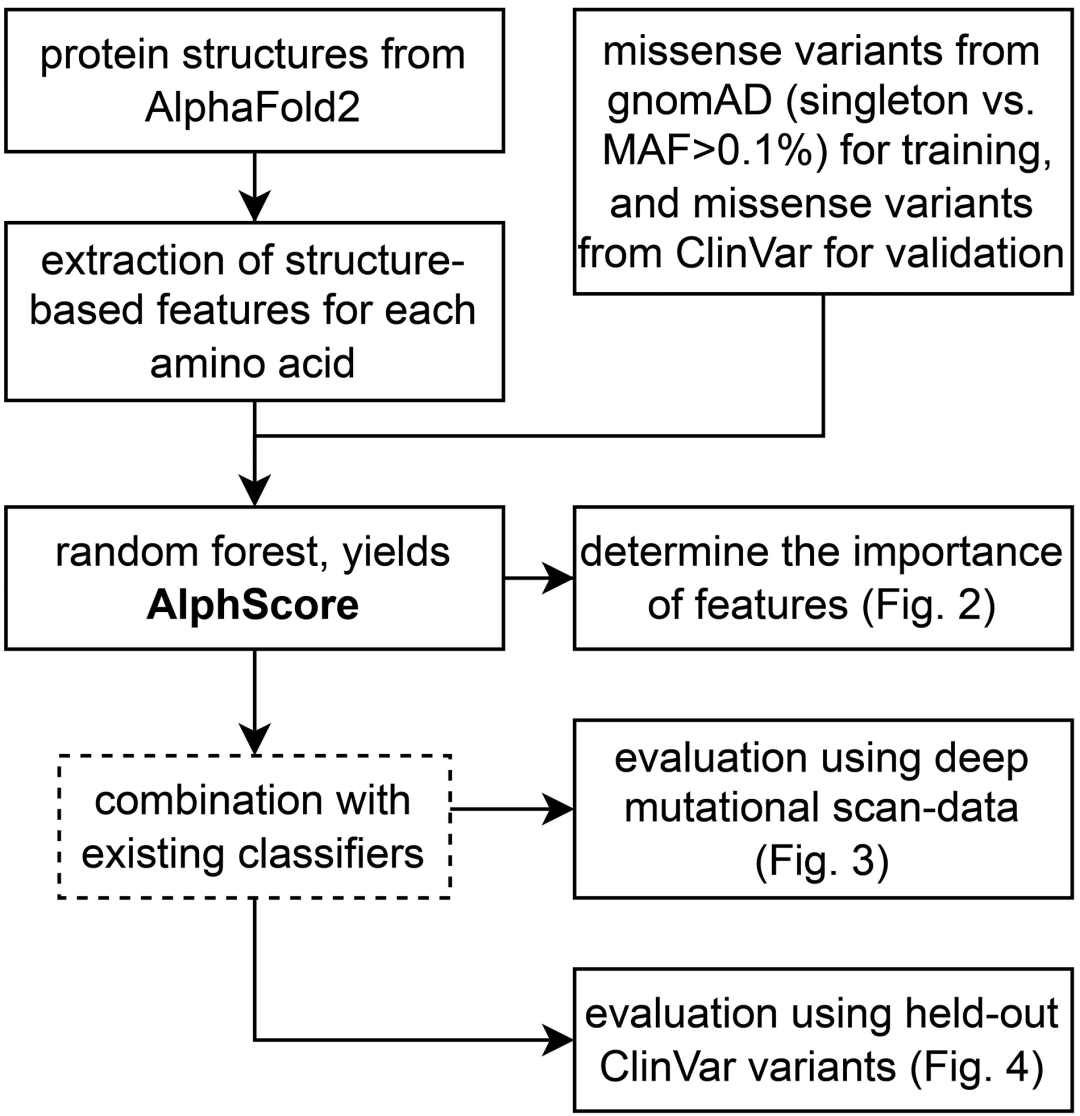
Workflow providing an overview of AlphScore generation and evaluation.

Figure 1 was created with the software draw.io (https://github.com/jgraph/drawio). Figures were assembled using Adobe Illustrator (version CS6).

## 3 Results

### 3.1 Generation of AlphScore

To determine the utility of AlphaFold2 structures for systematical prediction of the pathogenicity of missense variants, we followed the workflow shown in Fig. 1. First, structural features were extracted for each amino acid contained in AlphaFold2-predicted structures. To provide a comprehensive description of the environment of each amino acid in the structural models of AlphaFold2, 218 features such as molecular interactions, solvent accessibility, secondary structure, and amino acid network features were extracted (Supplementary Table S1). Second, the structural features of each amino acid were added to the dbNSFP database (version 4.2), which contains all potential non-synonymous single-nucleotide variants (SNVs) with extensive annotations (Liu et al. 2020). In total, structural features from 17 880 proteins were mapped to dbNSFP (listed in Supplementary Table S2). Third, three variant sets were created for training, validation, and testing, respectively, using the gnomAD and ClinVar databases (see Section 2). GnomAD variants were used as a training set (gnomAD_train, *n* = 315 876), since these variants are assumed to be less prone to biases such as curation, research history, clinical evaluation criteria, and existing computational missense prediction scores (Shah et al. 2018). GnomAD singleton variants and variants with a minor allele frequency (MAF) > 0.1% were used as proxy-pathogenic and proxy-benign variants, respectively (see Section 2 and Supplementary Fig. S2A for details on filter criteria). Independent sets of (probably) benign or (probably) pathogenic ClinVar missense variants were used for validation (ClinVar_val, total *n* = 35 640) and testing (ClinVar_test, total *n* = 21 068).

The gnomAD_train and the ClinVar_val sets of variants were used to train and evaluate three tree-based machine learning algorithms (i.e. gradient boosting, random forest, and extremely randomized trees), respectively. Hyperparameters were varied in a grid search. A model using random forests, termed AlphScore, achieved the best overall performance (AUROC of 0.793 on ClinVar_val, see Supplementary Table S3). To verify that AlphaFold2-based features play a genuine role in variant classification, a new model was fitted using the same algorithm following the removal of AlphaFold2-related features. This model (NullModel) retained only the reference and alternative amino acid as well as simple physicochemical properties of amino acids, and obtained a substantially lower AUROC (0.609). This demonstrates that AlphaFold2-based features are an important component of AlphScore. In the same analysis of the validation set, CADD achieved an AUROC of 0.871. However, AlphScore relies purely on AlphaFold2-based features, and does not contain features such as amino acid or nucleotide conservation. We also demonstrate that the results are robust toward slight changes in the selection criteria of proxy-pathogenic variants (Supplementary Fig. S2B).

### 3.2 Determination of most important features

**Figure 2.**
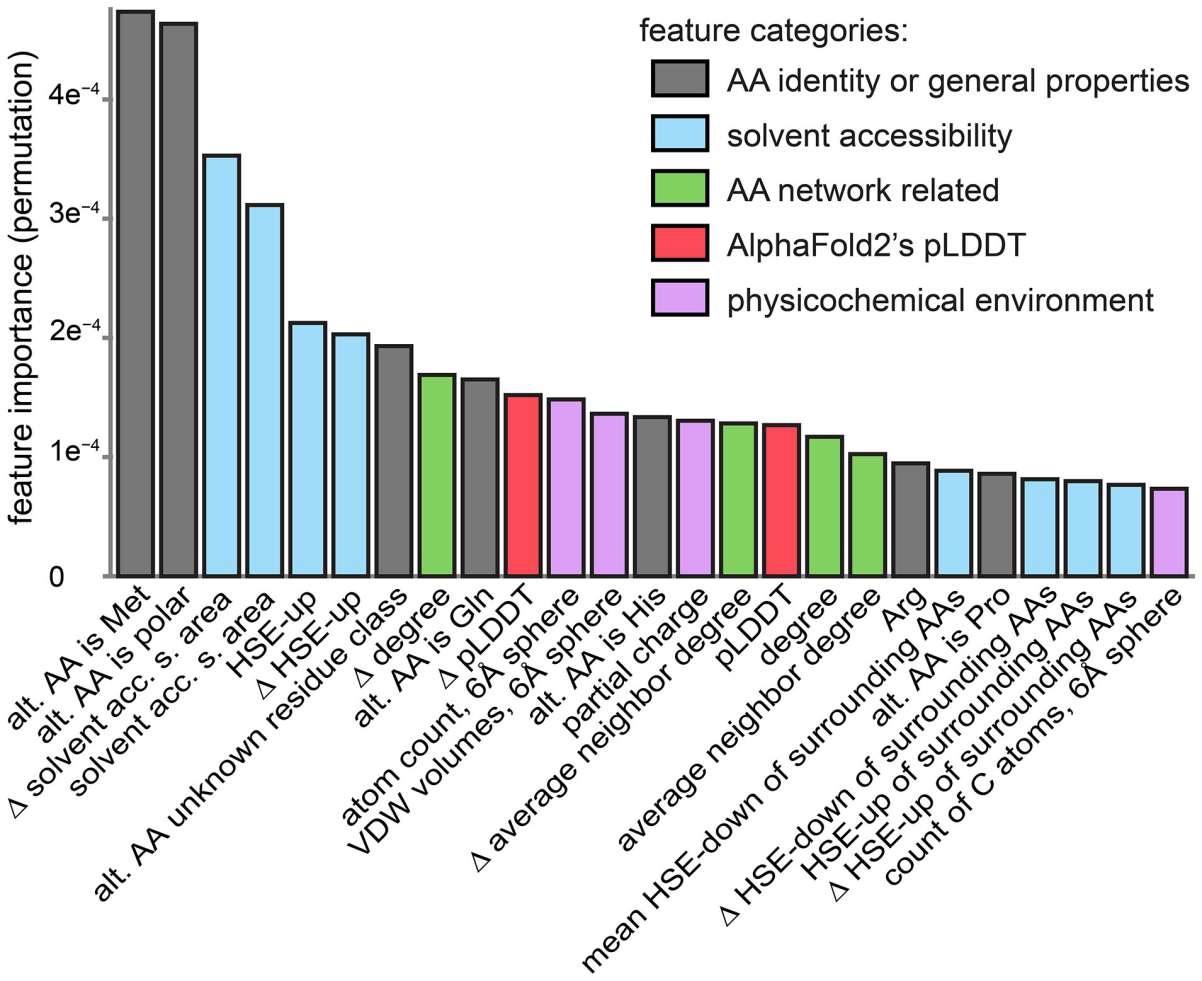
Importance of features in AlphScore. Bar graph displaying permutation-based feature importance for the 25 most important features (total number of features = 218). The feature categories are color-coded. The delta sign represents the difference between the alternative amino acid and the reference amino acid for a given feature (see Section 2). Alt. AA, alternative amino acid; AA, amino acid; Solvent acc. s. area, solvent accessible surface area; VDW, Van der Waals; HSE-up, half-sphere exposure of the upper sphere; HSE-down, half-sphere exposure of the lower sphere; pLDDT, predicted Local Distance Difference Test, AlphaFold2 per-residue confidence score.

To investigate which AlphaFold2-derived features were most important to AlphScore, permutation-based feature importances were calculated (Fig. 2 and Supplementary Table S4). Among the top 25 features, the most important AlphaFold2-based feature categories were: solvent accessibility (containing *n* = 8 features); amino acid network-related features (*n* = 4); features describing the physicochemical environment (*n* = 4); and AlphaFold2’s parameter for the reliability of its structural predictions, pLDDT (*n* = 2). The top 25 features included seven that were not derived from the structural models of AlphaFold2. Instead, these seven features described the identity or general properties of the reference or alternative amino acid.

Since the AlphaFold2 pLDDT is an interesting potential new parameter for predicting the pathogenicity of missense variants, pLDDT-based features were removed from the model on a test basis. As a result, the performance on the validation set (ClinVar_val) decreased marginally, from 0.793 to 0.790. Nevertheless, it seems interesting to note that both proxy-pathogenic gnomAD and (likely) pathogenic ClinVar variants tend to be located in protein regions with higher pLDDT values (see Supplementary Fig. S4).

### 3.3 Performance evaluation for AlphScore

To test AlphScore’s performance, AlphScore and the three *in silico* prediction scores were applied to missense variants from DMS or missense variants classified in ClinVar (ClinVar_test). In addition, combined scores of AlphScore and CADD, REVEL, and DEOGEN2, respectively, were created. To determine the best performing parameters for combination, logistic regression was used to fit ClinVar_val variants to combinations of scores (see Section 2).

**Figure 3.**
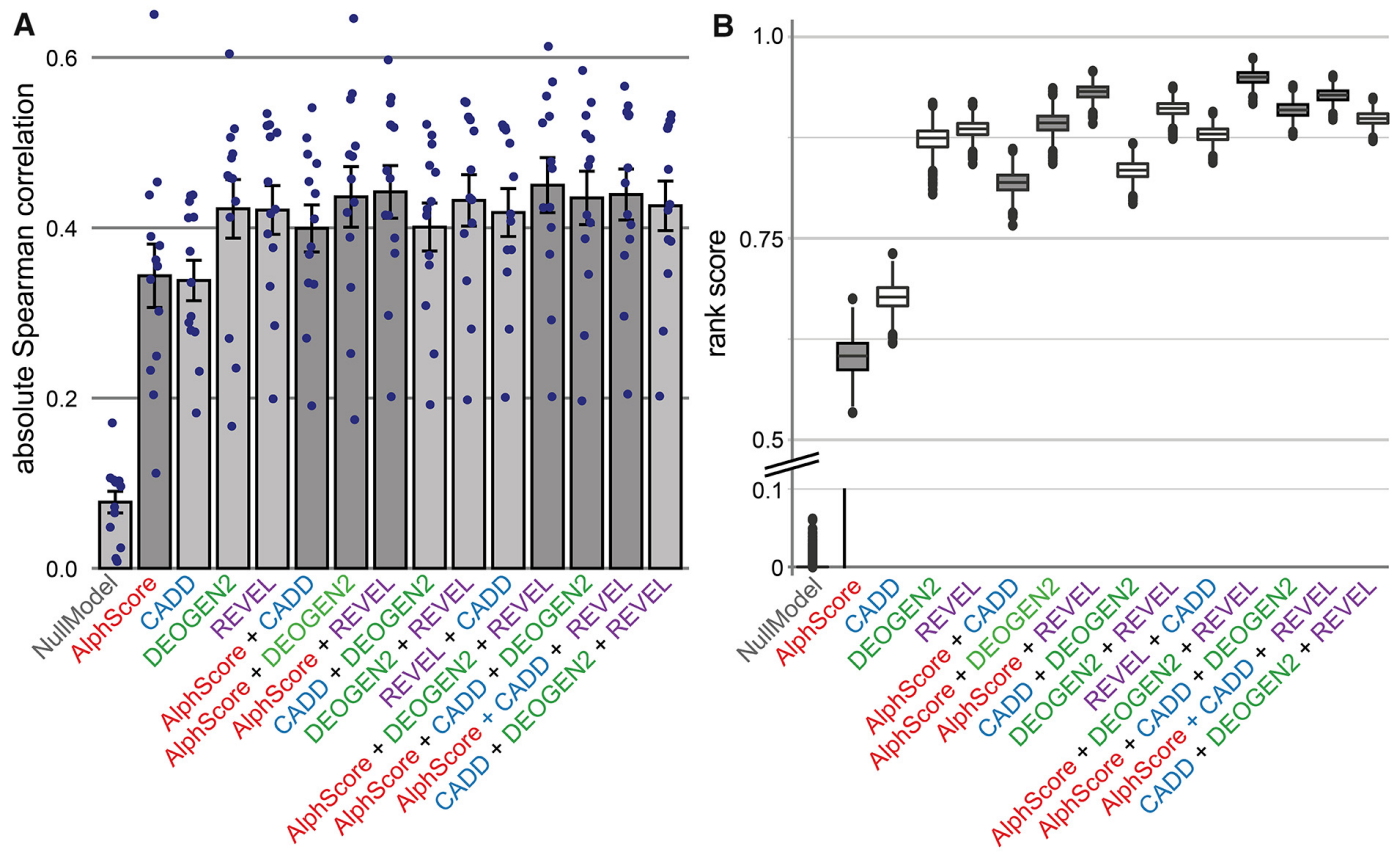
Combining AlphScore with established missense prediction scores improves correlation with DMS data. **(A)** Bar graphs displaying the mean absolute Spearman correlation between DMS scores and computational prediction scores. The dots represent the Spearman correlations of individual DMS experiments. The error bars represent the standard error of the mean. **(B)** Boxplots of rank scores as defined by Livesey and Marsh (2020), for 1000 bootstrapped samples. Higher rank scores indicate better Spearman correlations with DMS datasets compared with the competing prediction scores. The *y*-axis has been split to improve visibility. **(A** and **B)** Bars or box plots corresponding to scores containing AlphScore are highlighted in (darker) gray. Note that variants in the sets gnomAD_training and ClinVar_val were removed from the analyses.

DMS datasets that had been used in prior benchmarking analyses were used (Livesey and Marsh, 2020), and recent data on MSH2 and VKOR1 were added (Chiasson et al. 2020; Jia et al. 2021). In total, the present DMS dataset comprised 13 experiments in 11 proteins (ADRB2, BRCA1, HRAS, MSH2, P53, PTEN, SUMO1, TPK1, TPMT, UBE2I, and VKOR1). Calculations were then performed to determine the Spearman correlations between different computational prediction scores, and combinations thereof, and DMS scores. The average absolute Spearman correlation between AlphScore and the DMS scores was 0.344 (Supplementary Fig. S5). In comparison, the established scores achieved an average absolute Spearman correlation with the DMS scores of between 0.338 and 0.422 (Fig. 3A). For each prediction score, the addition of AlphScore increased the average Spearman correlation (CADD: 0.338–0.399; DEOGEN2: 0.422–0.436; REVEL: 0.421–0.442). Notably, while the highest overall Spearman correlation with the DMS data (0.450) was achieved by a combination of AlphScore with both DEOGEN2 and REVEL (Supplementary Fig. S6), it was not reached for the combination of the three existing prediction scores alone (Fig. 3). To investigate the reliability of those results, we applied bootstrapping (*n* = 1000) and calculation of the rank score, as introduced by Livesey and Marsh (2020; Fig. 3B). This rank score is calculated as average across proteins with a scale from 0 to 1. Prediction scores with higher correlations to DMS data receive a value closer to 1. Adding AlphScore improved the rank score for both CADD and REVEL in all and for DEOGEN2 in 95% of bootstrapped samples. The combination AlphScore with both DEOGEN2 and REVEL achieved the highest rank score in all bootstrapped samples.

**Figure 4.**
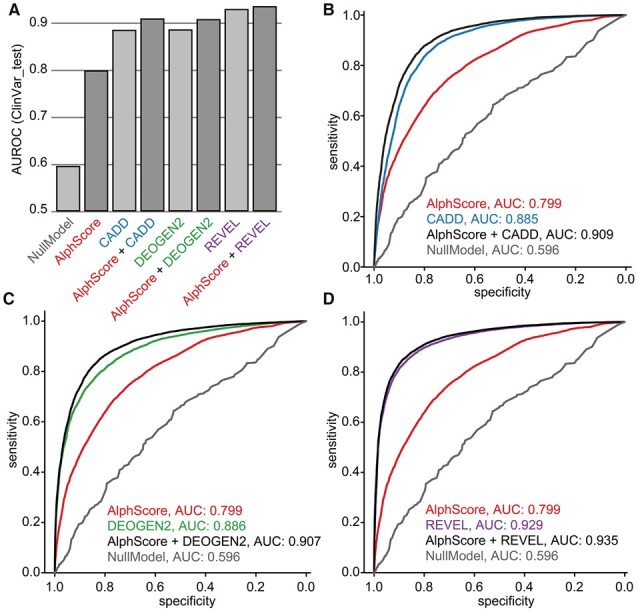
Combining AlphScore and established prediction scores improves the prediction of ClinVar variants. **(A)** Bar graphs representing the average AUROCs of the prediction scores denoted on the *x*-axis as obtained from the receiver operating characteristics (ROC) curves shown in B–D. Bars corresponding to scores containing AlphScore are highlighted in darker gray. **(B–D)** ROC curves showing the performance of AlphScore (B–D, red), CADD (B, blue), DEOGEN2 (C, green), REVEL (D, lilac) and linear combination of the AlphaFold-based score with the three existing prediction scores (black). A hold-out set of 9224 (likely) benign and 11 844 (likely) pathogenic missense variants from ClinVar (ClinVar_test) was used as data source. The gray line (NullModel) represents the baseline model, which was trained on the gnomAD training set, without AlphaFold-based features. AUC, area under the curve.

Investigations were then performed to determine how AlphScore and combinations of AlphScore with existing prediction scores would perform in the test set of ClinVar variants [ClinVar_test, *n* = 21 068, 9224 (likely) benign, 11 844 (likely) pathogenic], which contained proteins and variants that were independent from those contained in gnomAD_train and ClinVar_val (see Section 2). The AUROC of AlphScore alone was 0.799, whereas CADD, DEOGEN2, and REVEL achieved 0.885, 0.886, and 0.929, respectively. However, it should be noted that clinically relevant variants were included in the DEOGEN2 and REVEL training sets (variants from Humsavar/Uniprot or HGMD). Therefore, overlaps with ClinVar_test are likely, and reproduction of biases that may be present in ClinVar is possible. Again, in each case, the addition of AlphScore to the established scores increased the respective AUROC (CADD: 0.885–0.909; DEOGEN2: 0.886–0.907; and REVEL: 0.929–0.935; see Fig. 4). Use of the area under the curve in precision–recall curves as an alternative measure generated very similar results (Supplementary Fig. S7). Reassuringly, these results could be reproduced in all bootstrapped variant samples (*n* = 1000).

As an exploratory analysis we then divided our ClinVar_test dataset into two parts—variants located in proteins that are membrane-associated according to the PANTHER database (Thomas et al. 2003), and variants for which this is not the case (see Section 2). We determined the AUROC values for these two subsets of data, and no major differences were apparent (Supplementary Fig. S8).

### 3.4 Availability of pre-computed scores

Finally, the random forest was retrained on the complete gnomAD-derived training set with the aim to maximize power. Thus, no variants in specific proteins were held-out for testing. The resulting score was termed AlphScore_final and could be calculated for 80% (*n* = 66 931 527) of dbNSFP’s variants. Missing values are due to protein structures that could not be clearly mapped to dbNSFP. AlphScore_final and combinations of AlphScore_final with CADD, REVEL, or DEOGEN2 are available for download (DOI: 10.5281/zenodo.6288139; Supplementary Figs S9 and S10 and Supplementary Table S6 for additional data on the final scores).

## 4 Discussion

The present study generated AlphScore, a novel prediction score for missense variants which relies solely on features derived from the structural predictions of AlphaFold2. Comparisons with experimental high-throughput data and clinically informed variants from ClinVar showed that the addition of AlphScore improved the performance of established *in silico* missense prediction scores. This suggests that AlphScore captures information that is not encompassed in the existing scores, or that was sufficiently weighted in their training. To facilitate future work, the end-to-end pipeline and precomputed scores for 67 million missense variants have been made accessible to the wider research community.

When we analyzed structure-derived feature categories, we identified solvent accessibility as the most important feature category for AlphScore. This is in concordance with expectations, since the inaccessible core of a protein is strongly conserved (Overington et al. 1992), and disease associated missense variants are enriched in residues with low-solvent accessibility (Savojardo et al. 2020). Furthermore, among the 25 most important features we identified an interesting new parameter, i.e. the quality score of AlphaFold2, pLDDT, which, in addition to its technical sources, has been previously associated with intrinsically disordered protein regions (Ruff and Pappu 2021; Tunyasuvunakool et al. 2021). Regions with low pLDDT display a depletion of both pathogenic variants from ClinVar, and proxy-pathogenic variants from gnomAD. This could indicate that these regions tend to be less disease-relevant in aggregate than well-structured regions. However, intrinsically disordered regions were implicated in important functions, such as mediating interactions with protein domains or harboring posttranslational modifications (Babu 2016). In general, they also show lower conservation, as well as an altered amino acid composition, in comparison to well-structured regions (Brown et al. 2010), which could partially explain their depletion of pathogenic variants in ClinVar.

Despite the potential of AlphScore to improve existing missense prediction scores, several limitations in the current implementation of AlphScore remain. For instance, AlphScore only considers features that are represented in the structural environment of an amino acid in AlphaFold2 models. Thus, structural features such as posttranslational modifications, or features of the amino acid environment resulting from the quaternary structure, are not considered. Furthermore, since AlphScore is based solely on AlphaFold2 structural models, non-coding effects, such as alterations in splicing, are not considered either. Another limitation is proteins that are currently not covered by AlphScore, due to difficulties in mapping between Uniprot and genomic positions (https://www.uniprot.org/help/canonical_nucleotide).

To improve AlphScore, a number of avenues could be explored in future research. First, the choice of extracted features could be refined, or machine-learning algorithms that do not require manual feature engineering, such as 3D convolutional neural networks (Torng and Altman 2017), could be applied. Second, the training set might be optimized, e.g. by using variants with stronger signals of purifying selection [such as simulated *de novo* variants as opposed to proxy-pathogenic singleton variants (Kircher et al. 2014)]. Finally, additional layers of information, like species conservation or existing missense prediction scores, could be integrated into the score at an earlier stage, i.e. when training the initial random forest model. It will be interesting to see the relative importance of structure-based features in such integrated scores. This is particularly true if advanced models of sequence evolution, such as the recently published model EVE, are integrated with structural features, since AlphaFold2 structures themselves are based primarily on sequence evolution data. However, currently EVE only supports a limited set of genes (Frazer et al. 2021). Our opinion is that the implicit or explicit representation of human proteins as 3D structures will remain an important layer of information for missense variant interpretation.

The present study focused on predicting the pathogenicity of missense variants, rather than determining the pathogenic mechanism of a single variant. In principle, to investigate the pathogenic mechanism, allowing the AlphaFold2 model to predict the structure of the protein with the mutant amino acid sequence would be a logical approach. However, research has shown that AlphaFold2 does not accurately predict the structural effect of variants that are known to lead to a structural change (Buel and Walters 2022). At present, established methods such as molecular modeling should rather be considered to answer these questions. As input for such methods, and thus for the study of the pathogenic mechanism of missense variants, the AlphaFold2-based structures are a valuable resource.

In summary, we applied a machine-learning approach with classical feature extraction to the protein structures generated by AlphaFold2 and demonstrated that these structures contain information that is valuable in terms of predicting the pathogenicity of missense variants. We are eager to see how other groups will use AlphaFold2-based structures to predict the pathogenicity of missense variants and whether approaches, such as modifications of the AlphaFold2 model, might be competitive in predicting the pathogenicity of missense variants.

## Supplementary Material

btad280_Supplementary_Data

## Data Availability

AlphScore_final and combined scores computed in the present study have been submitted to Zenodo and are available under the DOI 10.5281/zenodo.6288139. The computational pipeline used to generate the data presented is available via github (github.com/Ax-Sch/AlphScore).
