# Supplementary material for: Predicting the pathogenicity of missense variants using features derived from AlphaFold2
Source: Bioinformatics. 2023 Apr 21;39(5):btad280. doi: 10.1093/bioinformatics/btad280 (PMC10203375; doi:10.1093/bioinformatics/btad280)
Supplement: btad280_Supplementary_Data [file btad280_supplementary_data.zip › Suppl_Figures.docx]

**Supplementary Figures**


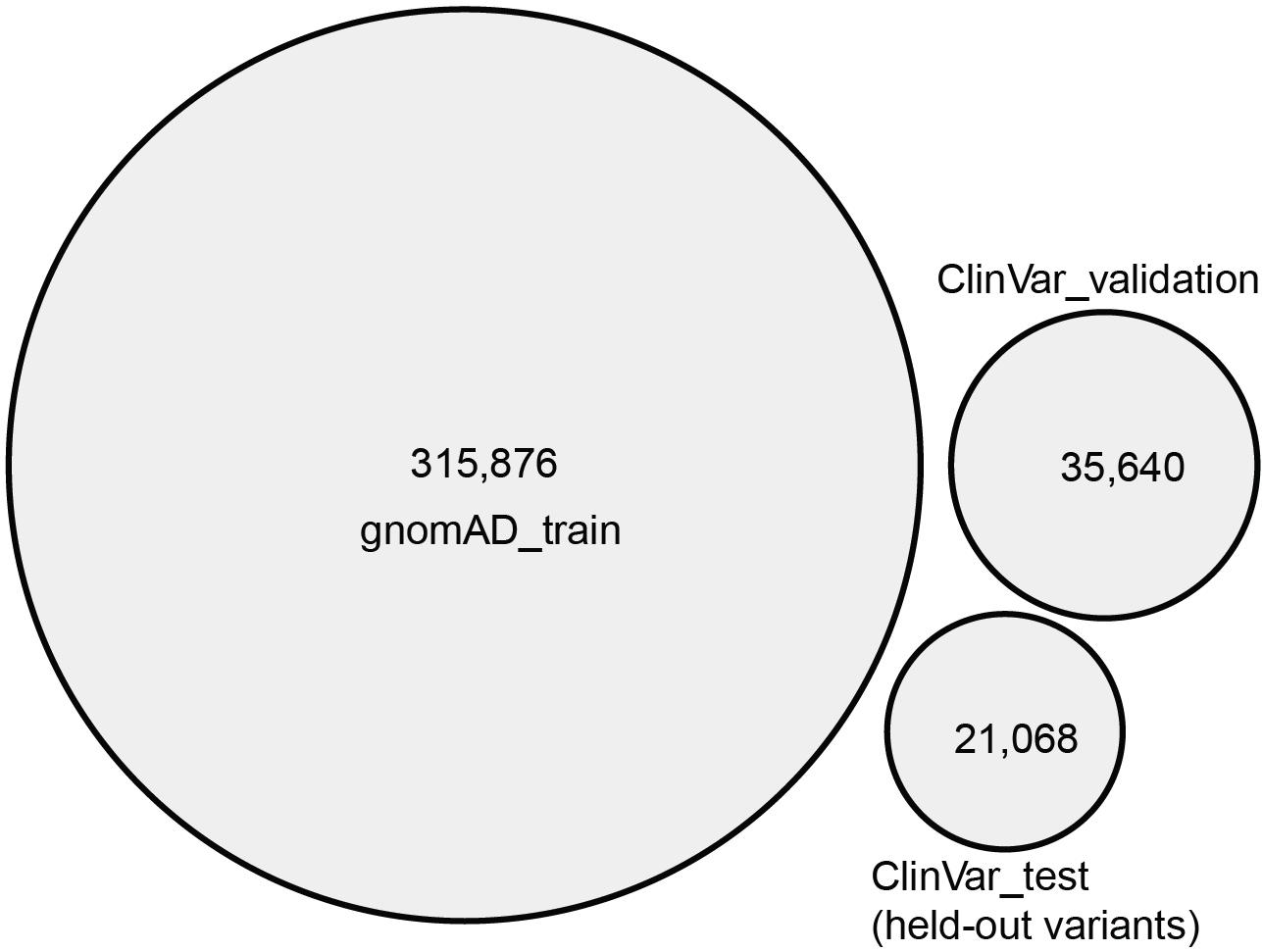


**Figure S1: Venn Diagram showing the variant sets used for training and testing.** Note that chromosome, genomic position reference and alternative allele were used to generate this plot, and that positions affecting more than one structure of AlphaFold2 are only counted once. Since the plot was generated prior to downsampling of gnomAD singleton variants, the final gnomAD training set is smaller. This plot illustrates that the training, validation and test datasets are non-overlapping. gnomAD_train: training set of gnomAD variants; ClinVar_validation: validation set of ClinVar variants; ClinVar_test: ClinVar variants that were held-out for testing.


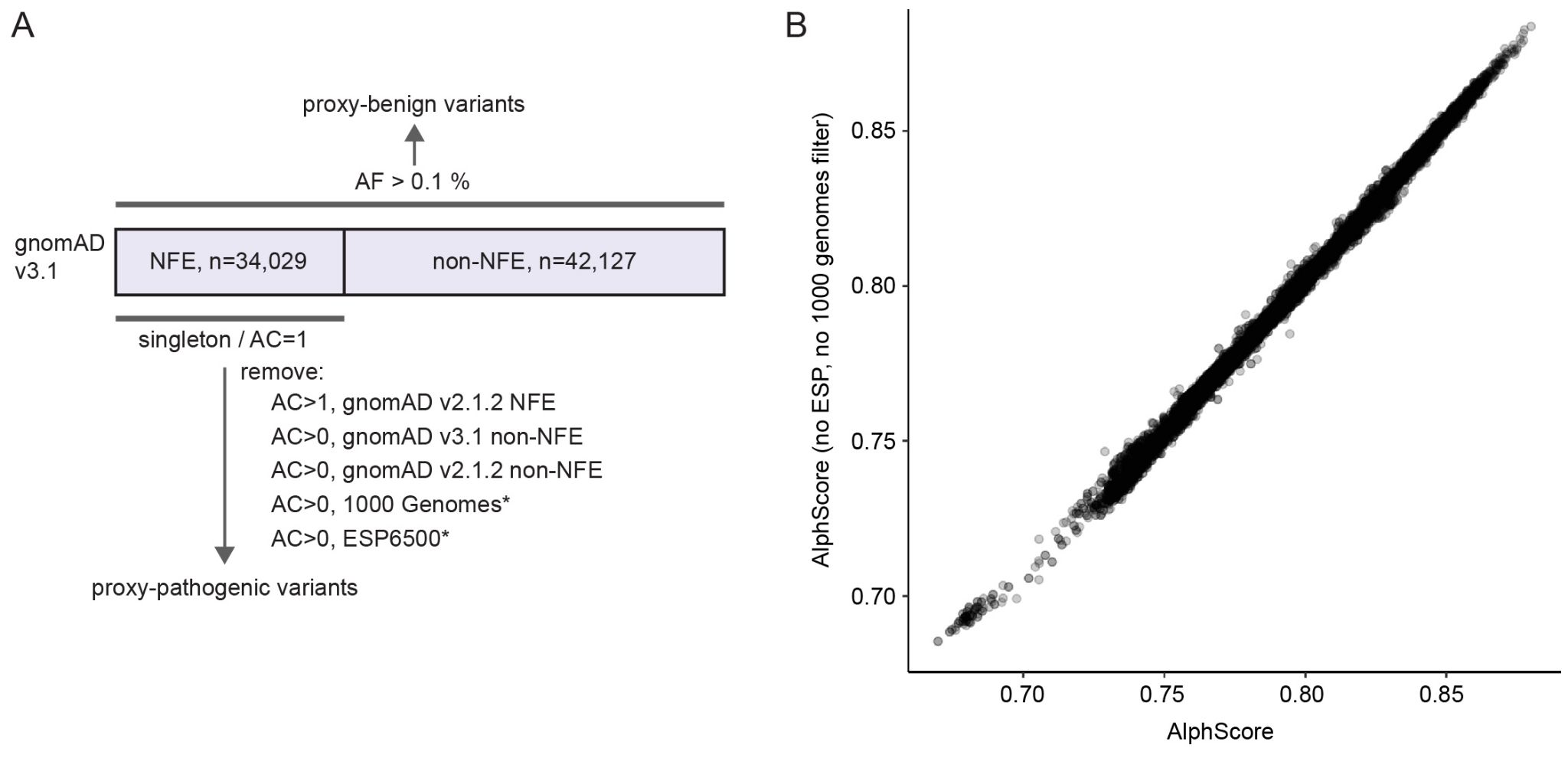


**Supplementary Figure S2: Construction of the training set and sensitivity of AlphScore to changes in the definition of proxy-pathogenic variants.** A) The flowchart shows the filter steps that were applied to create the gnomAD based training set. B) AlphScore values and values of a modified version of AlphScore are shown for a set of 16,407 ClinVar variants held out from training of both scores. In the modified version of AlphScore variants were not removed from the proxy-pathogenic set due to their presence in the ESP or the 1000 Genomes data set (the filters that have been omitted are marked with an asterisk in A). The Spearman correlation between both scores is 0.998. AC: Allele Count; AF: Allele Frequency; NFE: Non-Finnish-European; ESP6500: NHLBI Exome Sequencing Project (ESP) covering 6503 individuals, n: number of individuals.


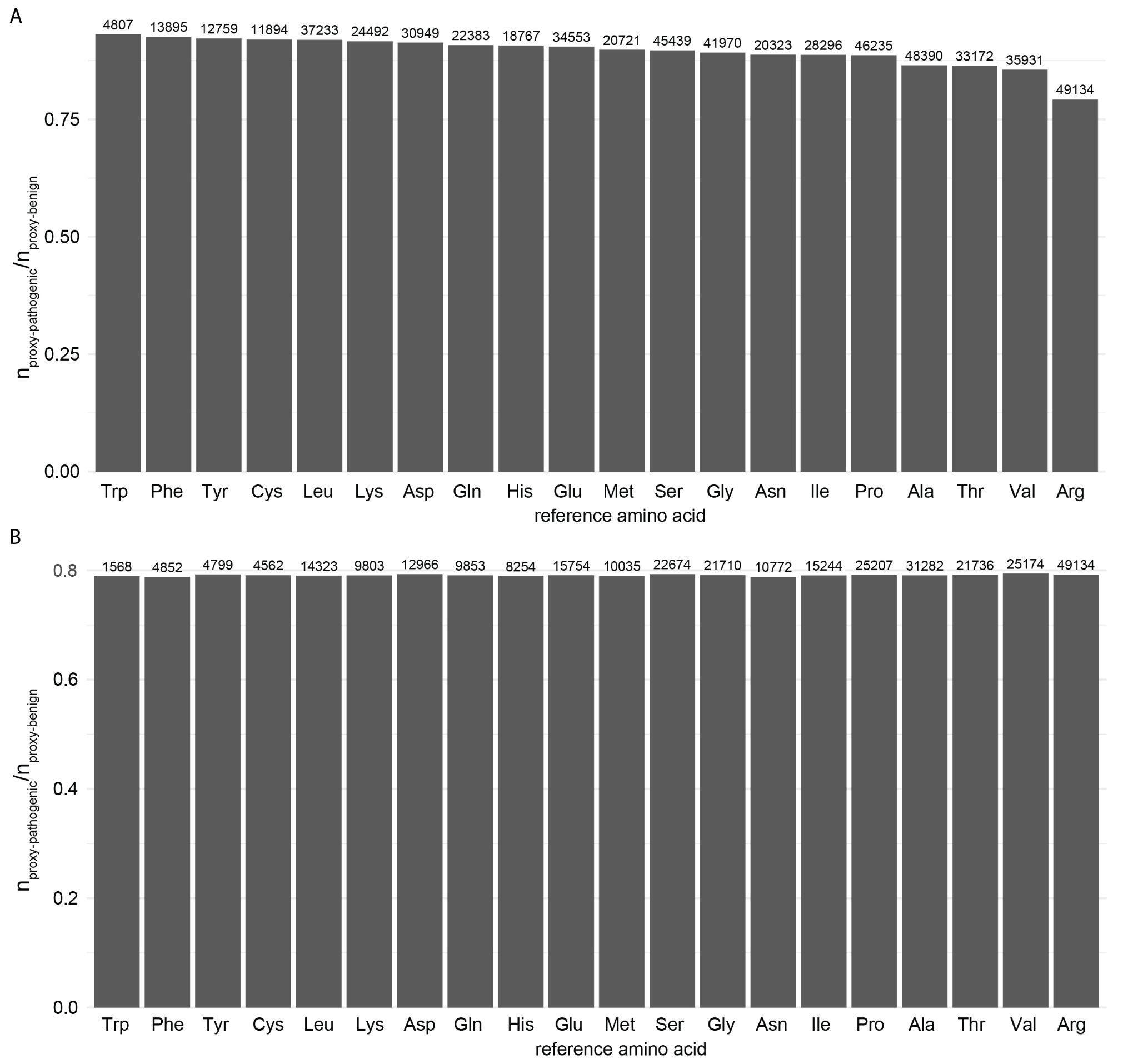


**Figure S3: Downsampling of proxy-pathogenic variants in gnomAD.** Bar graphs showing the ratios between proxy-pathogenic and proxy-benign variants before (A) and after (B) downsampling of proxy-pathogenic variants for each reference amino acid. The number above the bar corresponds to the total number of variants with the respective reference amino acid.

**
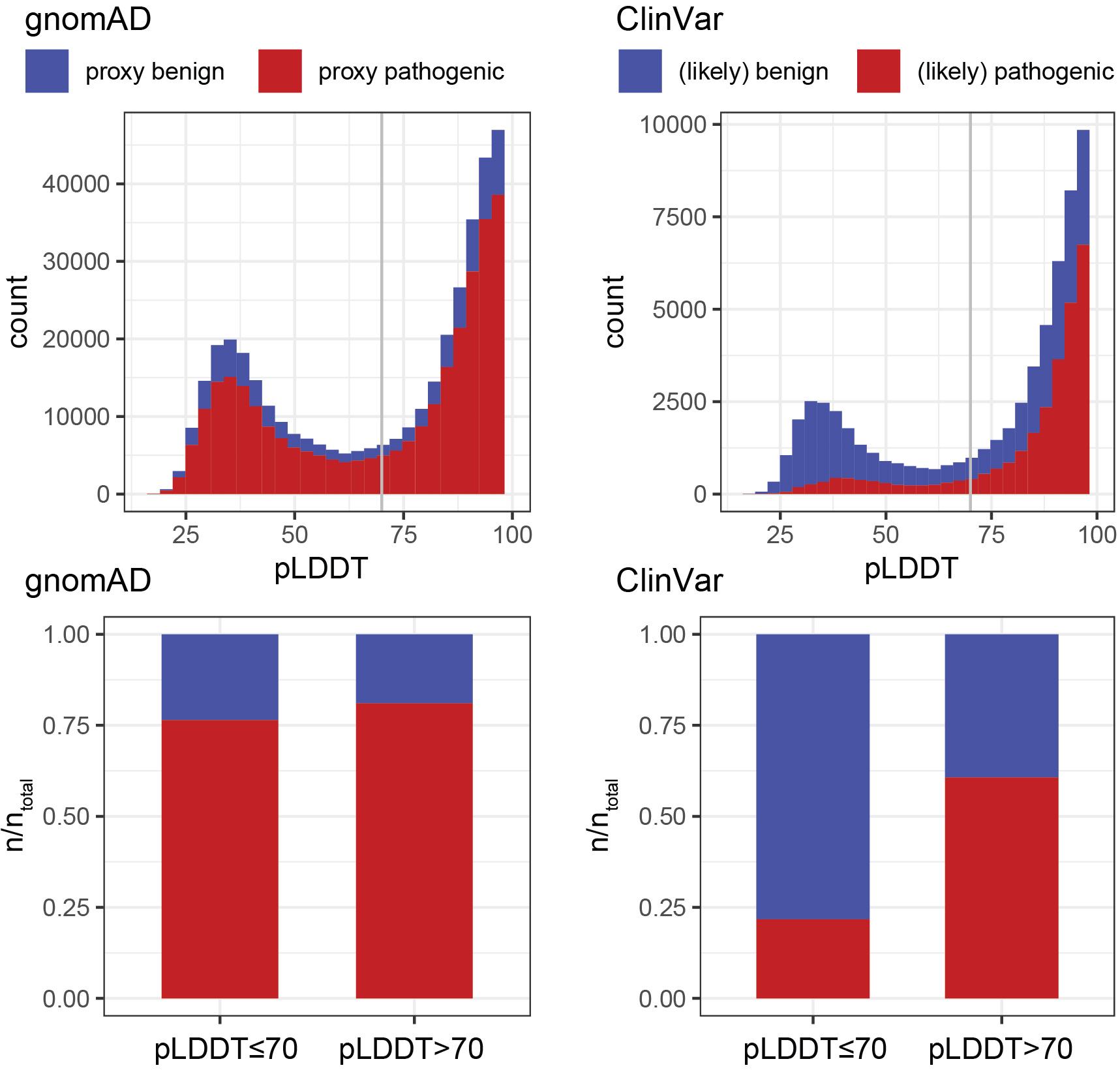
**

**Figure S4: The pLDDT score contains information of potential value in terms of the classification of missense variants.** The left panels contain data from the full gnomAD variant set, and the right panels contain data from the full ClinVar variant set (see Methods). The top row shows the distributions of pLDDT values of ClinVar or gnomAD variants as histograms. The histograms are colored according to the classification of the missense variants. The gray lines indicate a pLDDT of 70, since structural regions with pLDDT>70 are considered to be predicted with confidence. The bottom row shows the proportions of variant classifications, according to whether the pLDDT value at the respective position was above or below 70. pLDDT: predicted Local Distance Difference Test.


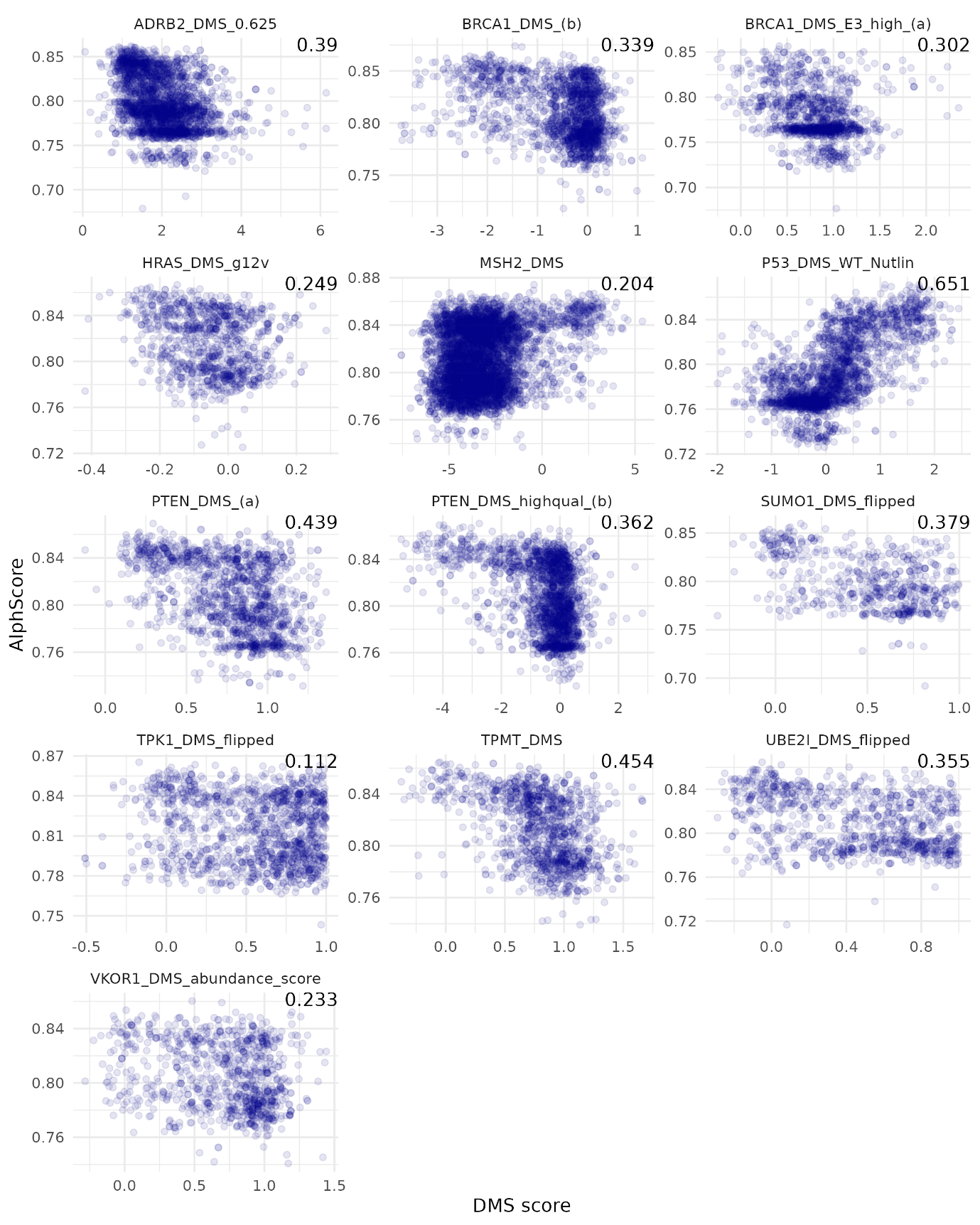


**Figure S5: Relationship between Deep Mutational Scan (DMS) data and AlphScore shown as scatter plots.** A separate panel is shown for each of the DMS experiments; the heading indicates the protein and the particular experiment. AlphScore is plotted on the y-axes and the values determined in DMS experiments are plotted on the x-axes. Each blue dot corresponds to a single missense-variant. The dots are shown semi-transparent for better visibility in case of overlapping dots. The values of the absolute Spearman correlations for each experiment are indicated at the top right, respectively.


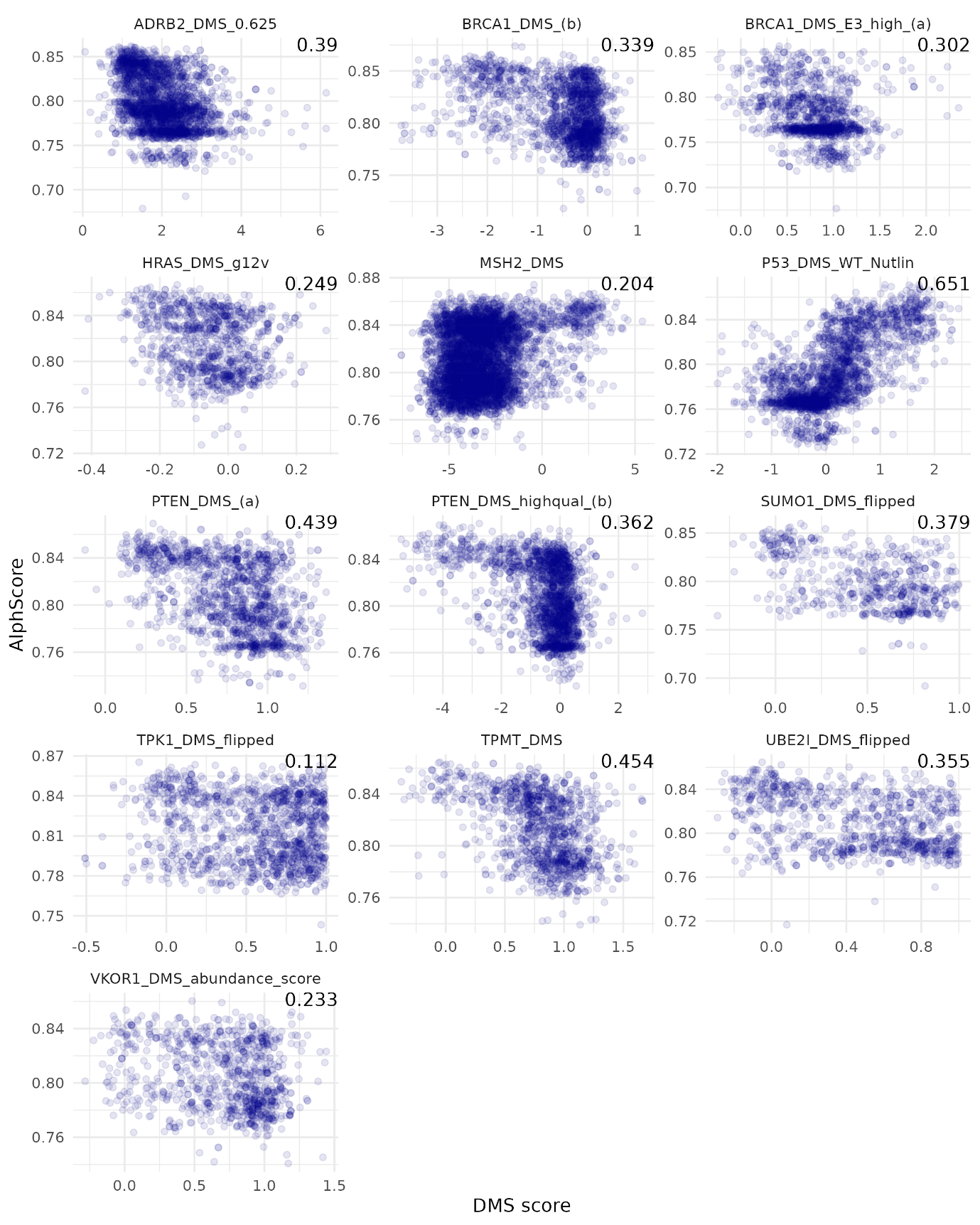


**Figure S6: Relationship between Deep Mutational Scan (DMS) data and the combination of AlphScore, DEOGEN2 and REVEL.** The figure is in analogy to Figure S5, except that the combination of AlphScore, DEOGEN2 and REVEL is shown on the y-axes.


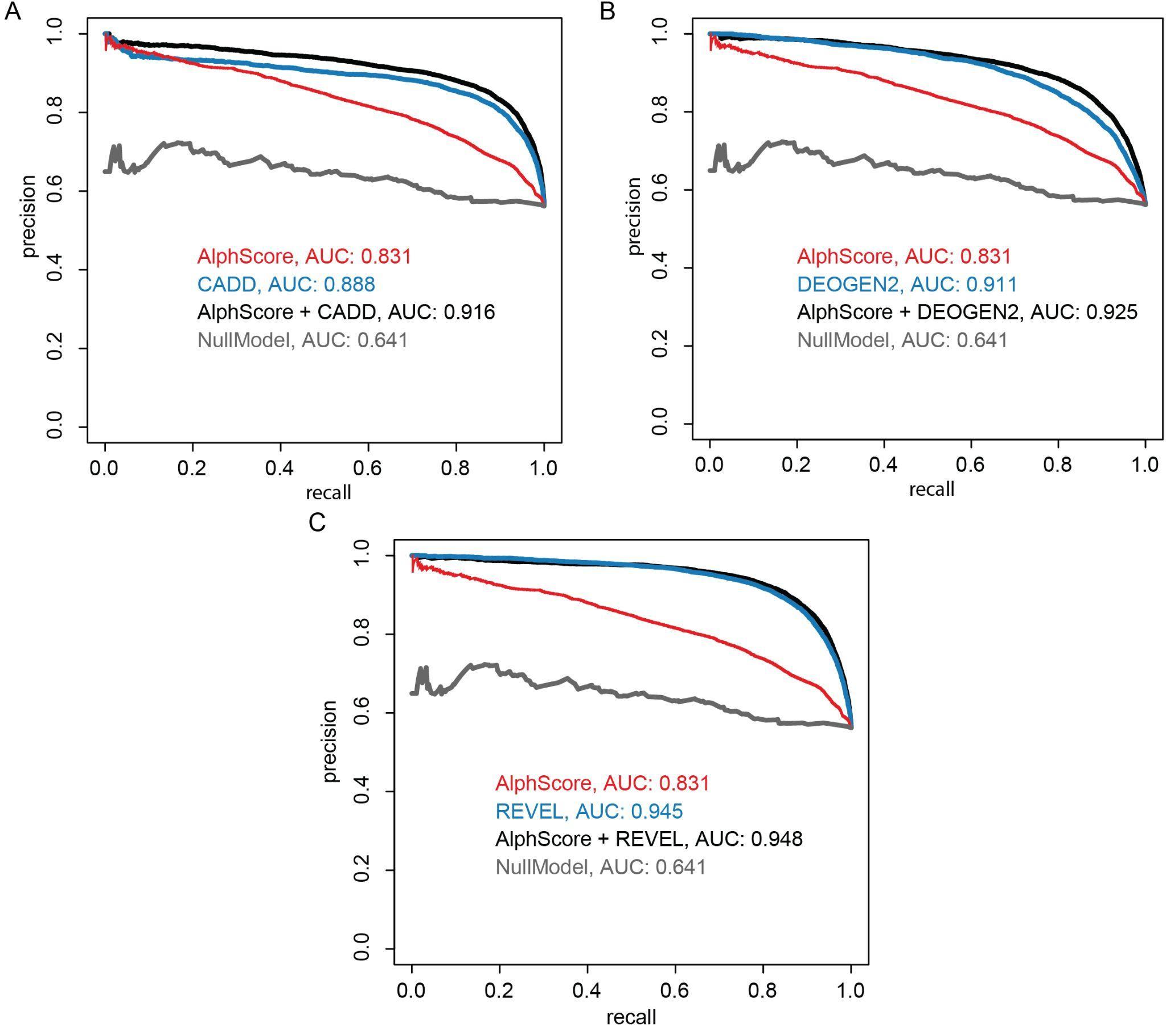


**Figure S7: Precision-Recall (PR) curves for AlphScore, established prediction scores, and combinations thereof.** The ClinVar variants (ClinVar_test) from Figure 4 were used as the data source. The diagrams were created using the pr.curve function of the R package PRROC. AUC: Area Under the Curve.


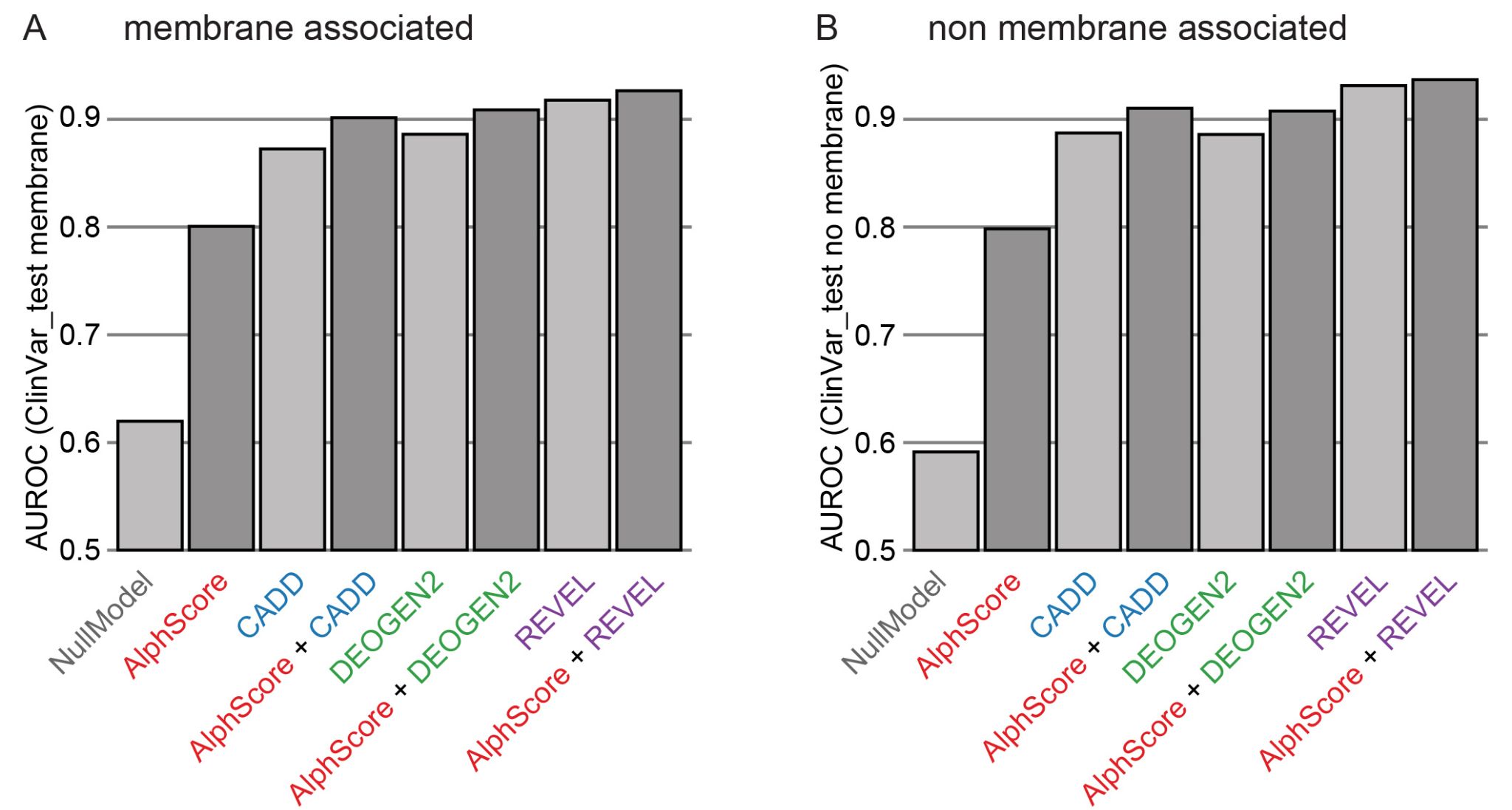


**Figure S8: Performance of AlphScore on membrane associated proteins.** (A and B) Bar graphs representing the average AUROCs of the prediction scores denoted on the x-axis as obtained from the Receiver Operating Characteristics (ROC) curves in analogy to Figure 4A. Bars corresponding to scores containing AlphScore are highlighted in (darker) gray. A hold-out set of 9,224 (likely) benign and 11,844 (likely) pathogenic missense variants from ClinVar (ClinVar_test) was used as data source and split into variants (n=3,815) in membrane associated proteins (A) and variants (n=17,253) not within membrane associated proteins (B) according to the PANTHER database. The list of protein classes used to define membrane associated proteins can be found in Table S5.

**
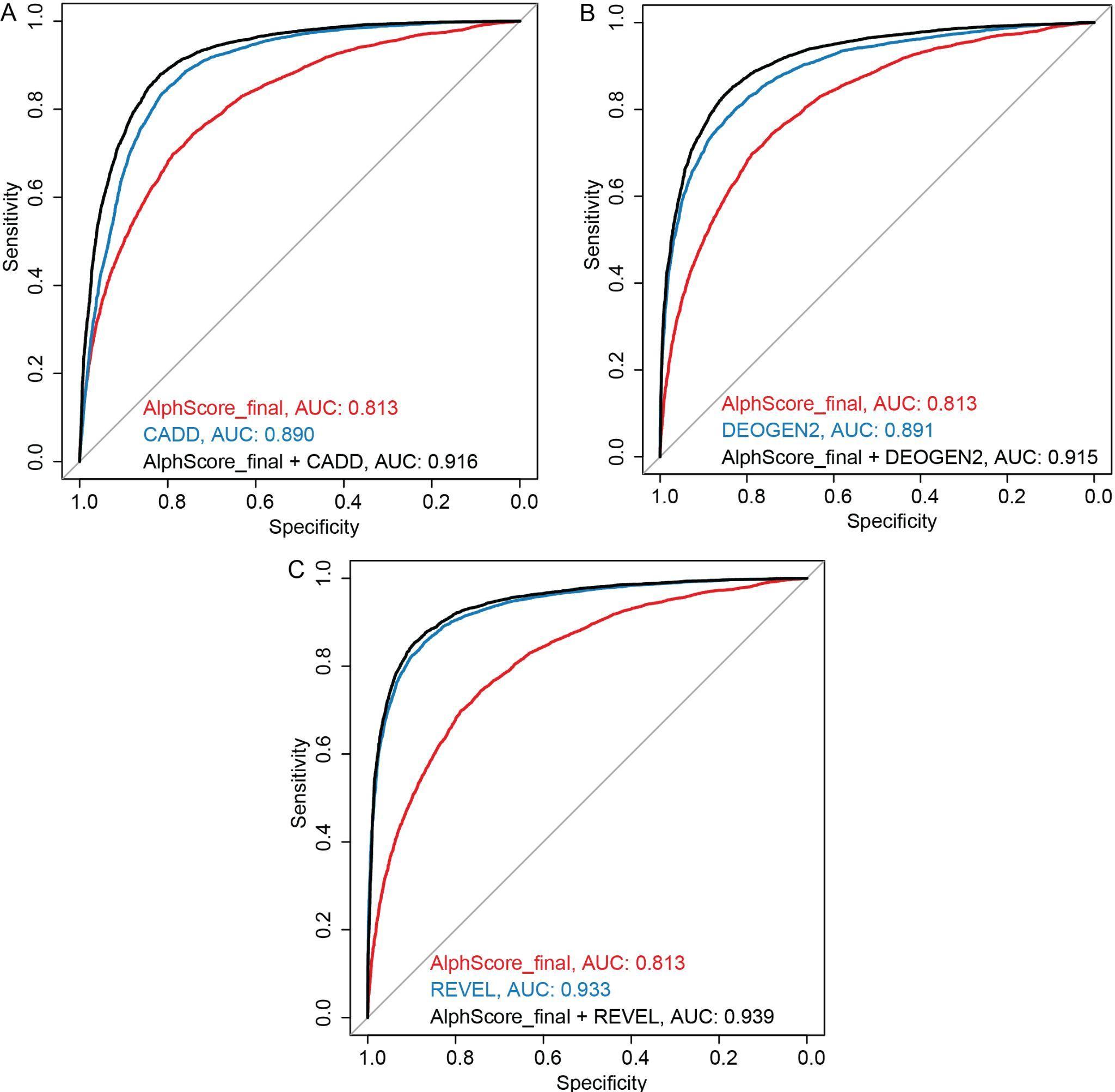
**

**Figure S9: Receiver Operating Characteristics (ROC) curves for AlphScore_final**. The ROC curves for (A) CADD, (B) DEOGEN2, (C) REVEL, and combinations thereof using logistic regression with AlphScore_final (A-C) are shown. The curves were generated using the subset of ClinVar_test that was new to ClinVar 20220109 and that was not used for training or validation of AlphScore_final or the combined scores. The diagrams were created using the roc function of the R package pROC. AUC: Area Under the Curve.


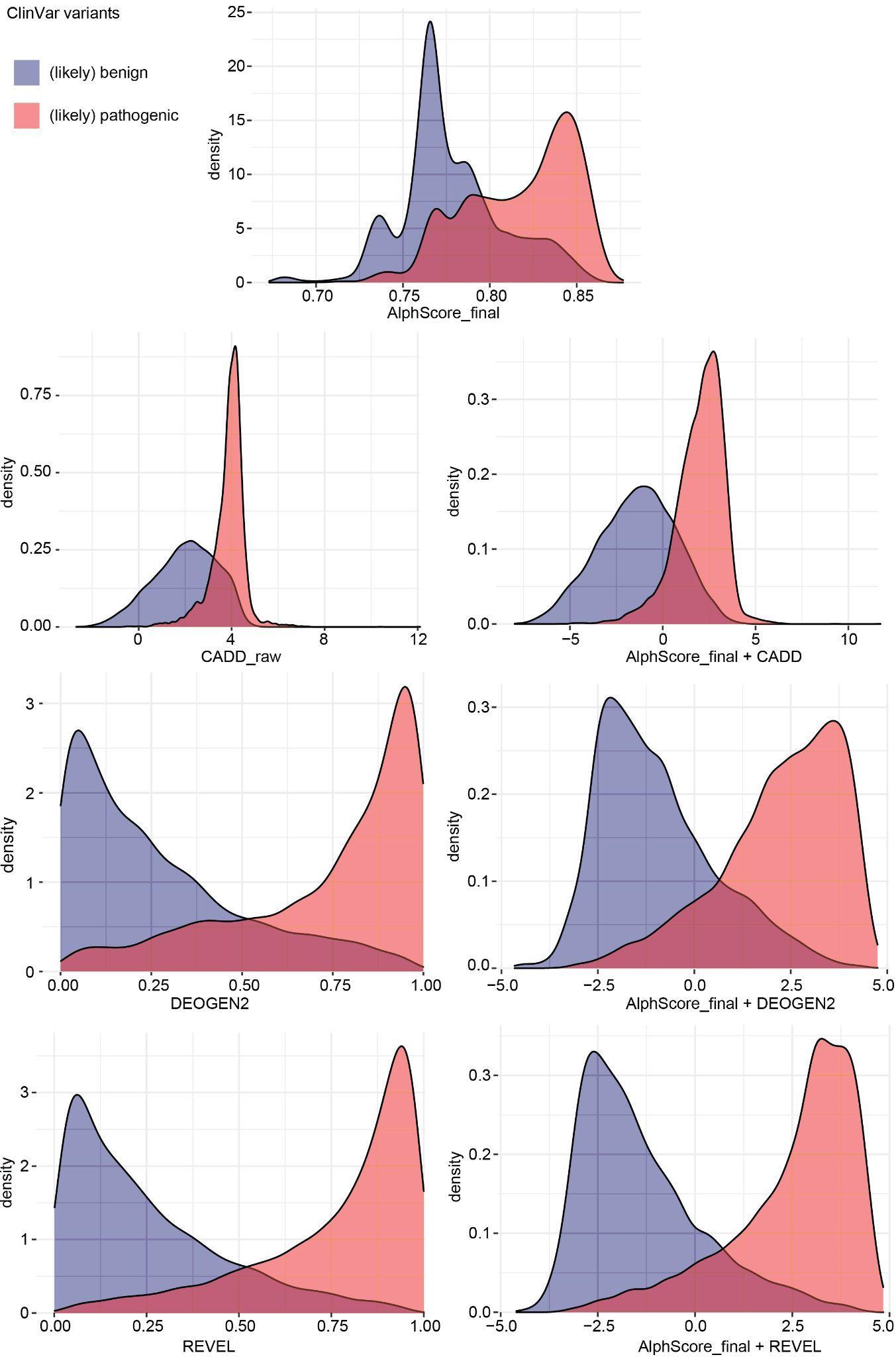


**Figure S10: Smoothed density curves of the final predictors using ClinVar variants.** A subset of ClinVar_test that was new to ClinVar 20220109 and that was not used for training or validation of AlphScore_final or the combined scores was used as data source. The x-axis shows the values of the respective scores. The y-axis shows the density of the (likely) benign (blue) and the (likely) pathogenic (red) variants, respectively. The plots were generated using the geom_density function of the ggplot2 package in R with default parameters.
